# Characterizing the Fused TvG6PD::6PGL Protein from the Protozoan *Trichomonas vaginalis*, and Effects of the NADP^+^ Molecule on Enzyme Stability

**DOI:** 10.3390/ijms21144831

**Published:** 2020-07-08

**Authors:** Laura Morales-Luna, Beatriz Hernández-Ochoa, Edson Jiovany Ramírez-Nava, Víctor Martínez-Rosas, Paulina Ortiz-Ramírez, Fabiola Fernández-Rosario, Abigail González-Valdez, Noemí Cárdenas-Rodríguez, Hugo Serrano-Posada, Sara Centeno-Leija, Roberto Arreguin-Espinosa, Miguel Cuevas-Cruz, Daniel Ortega-Cuellar, Verónica Pérez de la Cruz, Luz María Rocha-Ramírez, Edgar Sierra-Palacios, Rosa Angélica Castillo-Rodríguez, Vanesa Vega-García, Yadira Rufino-González, Jaime Marcial-Quino, Saúl Gómez-Manzo

**Affiliations:** 1 Laboratorio de Bioquímica Genética, Instituto Nacional de Pediatría, Secretaría de Salud, 04530 Ciudad de México, Mexico; lauraeloisamorales@ciencias.unam.mx (L.M.-L.); edsonjiovany@ciencias.unam.mx (E.J.R.-N.); ing_vicmr@hotmail.com (V.M.-R.); paulo.r1396@gmail.com (P.O.-R.); faby.fernandez.ross@gmail.com (F.F.-R.); 2 Posgrado en Ciencias Biológicas, Universidad Nacional Autónoma de México, 04510 Ciudad de México, Mexico; 3 Laboratorio de Inmunoquímica, Hospital Infantil de México Federico Gómez, Secretaría de Salud, 06720 Ciudad de México, Mexico; beatrizhb_16@comunidad.unam.mx; 4 Programa de Posgrado en Biomedicina y Biotecnología Molecular, Escuela Nacional de Ciencias Biológicas, Instituto Politécnico Nacional, 11340 Ciudad de México, Mexico; 5 Departamento de Biología Molecular y Biotecnología, Instituto de Investigaciones Biomédicas, Universidad Nacional Autónoma de México, 04510 Ciudad de México, Mexico; abigaila@biomedicas.unam.mx; 6 Laboratorio de Neurociencias, Instituto Nacional de Pediatría, Secretaría de Salud, 04530 Ciudad de México, Mexico; noemicr2001@yahoo.com.mx; 7 Consejo Nacional de Ciencia y Tecnología (CONACYT), Laboratorio de Agrobiotecnología, Tecnoparque CLQ, Universidad de Colima, Carretera los Limones-Loma de Juárez, 28629 Colima, Mexico; hjserranopo@conacyt.mx (H.S.-P.); scenteno0@ucol.mx (S.C.-L.); 8 Departamento de Química de Biomacromoléculas, Instituto de Química, Universidad Nacional Autónoma de México, 04510 Ciudad de México, Mexico; arrespin@unam.mx (R.A.-E.); miguel.ccqi@yahoo.com.mx (M.C.-C.); 9 Laboratorio de Nutrición Experimental, Instituto Nacional de Pediatría, 04530 Secretaría de Salud, Mexico; dortegadan@gmail.com; 10 Departamento de Neuroquímica, Instituto Nacional de Neurología y Neurocirugía Manuel Velasco Suárez, Secretaria de Salud, 14269 Ciudad de México, Mexico; veped@yahoo.com.mx; 11 Unidad de Investigación en Enfermedades Infecciosas, Hospital Infantil de México Federico Gómez, Dr. Márquez No. 162, Col Doctores, 06720 Delegación Cuauhtémoc, Mexico; luzmrr7@yahoo.com.mx; 12 Colegio de Ciencias y Humanidades, Plantel Casa Libertad, Universidad Autónoma de la Ciudad de México, 09620 Ciudad de México, Mexico; edgar.sierra@uacm.edu.mx; 13 Consejo Nacional de Ciencia y Tecnología (CONACYT), Instituto Nacional de Pediatría, Secretaría de Salud, 04530 Ciudad de México, Mexico; racastilloro@conacyt.mx; 14 Facultad de Ciencias, Universidad Nacional Autónoma de México, 04510 Ciudad de México, Mexico; e-vanessavg@gmail.com; 15 Laboratorio de Parasitología Experimental, Instituto Nacional de Pediatría, Secretaría de Salud, 04530 Ciudad de México, Mexico; yadirg@gmail.com

**Keywords:** *Trichomonas vaginalis*, heterologous expression, G6PD, biochemical characterization, 3D-structure

## Abstract

This report describes a functional and structural analysis of fused glucose-6-phosphate dehydrogenase dehydrogenase-phosphogluconolactonase protein from the protozoan *Trichomonas vaginalis* (*T. vaginalis*). The glucose-6-phosphate dehydrogenase (*g6pd*) gene from *T*. *vaginalis* was isolated by PCR and the sequence of the product showed that is fused with *6pgl* gene. The fused Tv*g6pd*::*6pgl* gene was cloned and overexpressed in a heterologous system. The recombinant protein was purified by affinity chromatography, and the oligomeric state of the TvG6PD::6PGL protein was found as tetramer, with an optimal pH of 8.0. The kinetic parameters for the G6PD domain were determined using glucose-6-phosphate (G6P) and nicotinamide adenine dinucleotide phosphate (NADP^+^) as substrates. Biochemical assays as the effects of temperature, susceptibility to trypsin digestion, and analysis of hydrochloride of guanidine on protein stability in the presence or absence of NADP^+^ were performed. These results revealed that the protein becomes more stable in the presence of the NADP^+^. In addition, we determined the dissociation constant for the binding (*K_d_*) of NADP^+^ in the protein and suggests the possible structural site in the fused TvG6PD::6PGL protein. Finally, computational modeling studies were performed to obtain an approximation of the structure of TvG6PD::6PGL. The generated model showed differences with the GlG6PD::6PGL protein (even more so with human G6PD) despite both being fused.

## 1. Introduction

*Trichomonas vaginalis* is a unicellular and anaerobic pathogenic protozoon facultative that reproduces by binary fusion [1]. This parasite causes the most common human non-viral sexually transmitted infection worldwide, known as trichomoniasis [2,3,4], which causes about 250 million infections each year [1]. *T. vaginalis* is a parasite that colonizes the urogenital tract of both women and men [1,4], although it has also been detected and isolated from the respiratory tracts of infants [5] and adults [6]. In women, this pathogen adheres to and damages vaginal epithelial cells and causes urethritis, vaginitis, and cervicitis [1], causing symptoms ranging from a relatively asymptomatic state to severe inflammation [7]. In addition, infected women have several complications associated with unfavorable pregnancy outcomes, such as premature delivery, low birth weight [8], a greater risk of HIV transmission and/or acquisition [9], and cervical cancer [10]. In men, the symptoms and prevalence of trichomoniasis are less well characterized, but the infection appears to be asymptomatic in 70% of male cases [11].

This parasite uses carbohydrates as its main energy source through its fermentative metabolism under aerobic and anaerobic conditions, which produces acid end products [12]. The metabolic pathways of this parasite share similar characteristics with eukaryotes and anaerobic prokaryotes, but *T*. *vaginalis* does not have the ability to synthesize macromolecules de novo since it lacks the enzymes necessary for the synthesis of purine, pyrimidine, and some lipids as cholesterol; which are acquired from vaginal secretions or through the phagocytosis of host and bacterial cells [13,14].

This pathogen belongs to the parabasal lineage of microaerophilic eukaryotes that do not have mitochondria or peroxisomes but do possess organelles called hydrogenosomes, a structure where molecular hydrogen and adenosine triphosphate (ATP) are produced. Indeed, there is phylogenetic and biochemical evidence that indicates these hydrogenosomes have a common origin with the mitochondria [14]. Since *T. vaginalis* is a microaerophilic microorganism, it requires redox and antioxidant systems to counter the harmful effects of oxygen and to express a wide range of genes encoding for defense molecules, including peroxiredoxins, thioredoxin reductases, superoxide dismutase, and rubrerythrin [15]. Two main activities have been identified, whereby NADPH oxidase and NADH oxidase eliminate oxygen present in the cytoplasm [16].

Another important metabolic pathway in the life-cycle of these parasites is the pentose phosphate pathway (PPP), which consists of an oxidative and non-oxidative phase. In the oxidative phase, the glucose-6-phosphate dehydrogenase (G6PD) is the first enzyme that catalyze an irreversible reaction of glucose-6-phosphate (G6P) to 6-phosphoglucono-δ-lactone with the production of the first reduced form of nicotinamide adenine dinucleotide phosphate (NADPH) molecule; while that the second enzyme, the 6-phosphogluconolactonase (6PGL), catalyzes the hydrolysis of 6-phosphoglucono-δ-lactone to produce 6-phosphogluconate, and finally the 6-phosphogluconate dehydrogenase (6PGD) enzyme decarboxylated to 6-phosphogluconate to yield a second NADPH molecule and one molecule of five-carbon atoms ribulose 5-phosphate (Ru5P), which enters the non-oxidative phase (Supplementary Materials Figure S1). The PPP provides a wide variety of fundamental molecules, such as NADPH, which serves as a hydrogen donor molecule in biosynthetic processes. It may also play an important role in the parasite in defending against the oxidative attack of the infected host. Further, the PPP also provides ribose 5-phosphate, which functions as a precursor to nucleic acids and the metabolic intermediates of glycolysis [17]. Considering the role of glucose-6-phosphate dehydrogenase (G6PD) in metabolism, this enzyme has been considered as a pharmacological target for some microorganisms, such as the protozoan that causes malaria *Plasmodium falciparum* [18]. The G6PD protein from *T. vaginalis* (TvG6PD) differs in its length and sequence from human G6PD because the *g6pd* gene is fused with 6-phosphogluconolactonase (*6pgl*) gene [19], which causes the structure of both proteins to be different. Due to binding of the 6PGL sequence to G6PD, the entire protein has no sequence homology to humans and other mammalian genes, analyzing the G6PD of *T. vaginalis* can allow us to determine its functional and structural properties; these properties seem to offer important potential for design of inhibitors on G6PD of *T. vaginalis*, which could selectively block the PPP metabolic pathway of the parasite.

In this study, the *g6pd* gene from *T*. *vaginalis* was isolated, cloned, and expressed in a bacterial system. Although the fused protein (TvG6PD::6PGL) was purified, only the kinetic values of the G6PD protein were determined. With the recombinant protein, it was also possible to obtain its structural parameters, and a three-dimensional (3D) model was generated to determine more details about the structure of the TvG6PD::6PGL. This is the first study on the characterization of a protein that participates in the PPP of *T*. *vaginalis* to propose it as a therapeutic target for the analysis (or development) of specific drugs and that serves to control this pathogen that is of clinical relevance to humans.

## 2. Results and Discussion

### 2.1. Cloning of TvG6PD::6PGL

**Figure 1:**
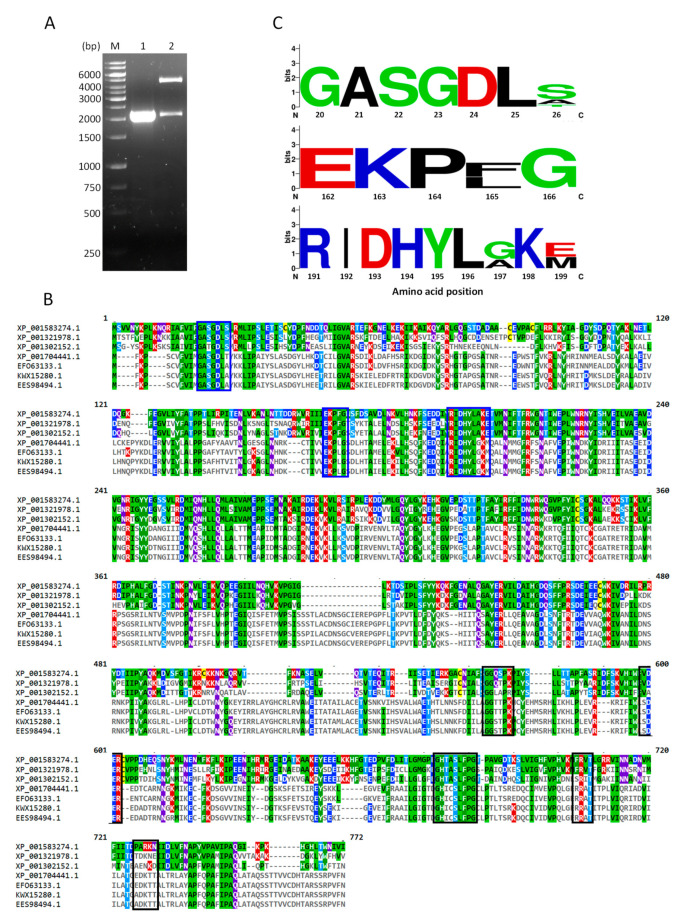
Cloning the glucose-6-phosphate dehydrogenase-phosphogluconolactonase (*g6pd::6pgl*) gene from *T. vaginalis* and an analysis of the protein sequence. (**A**) Agarose gel (1%) showing the amplification of the *TvG6PD::6PGL* gene by PCR (lane 1) and verifying the cloning of the gene in the expression vector (pET3a-HisTEV) by digestion with the restriction enzyme *Nde*I-*Bam*HI (lane 2). M: The GeneRuler 1 kb DNA marker (Thermo Scientific, Waltham, MA, USA). (**B**) Multiple sequence alignment of the amino acids of G6PD::6PGL between *T. vaginalis* and *G. lamblia*. The sequences of G6PDs used for alignment were XP_001583274.1 (*Trichomonas vaginalis* G3), XP_001321978.1 (*T. vaginalis* G3), XP_001302152.1 (*T. vaginalis* G3), XP_001704441.1 (*Giardia lamblia* ATCC 50803), KWX15280.1 (*G. intestinalis* assemblage B, EFO63133.1 (*G. lamblia* P15), and EES98494.1 (*G. intestinalis* ATCC 50581). The fragments conserved for G6PD are indicated in blue boxes and those for the 6PGL are shown in black boxes. The percentage of amino acid identity found between the *T. vaginalis* (TvG6pd::6PGL) sequence with respect to the *G*. *lamblia* sequences was 30%, even though both proteins are fused. Multiple sequence alignment was done with ClustalW and visualized with MView. (**C**) Comparison of conserved sequences between *Giardia*, *Trichomonas*, and *Homo sapiens* G6PD made with the weblogo online program.

As reported for the *T. vaginal* genome, the *g6pd* gene is fused with the *6-phosphogluconolactonase* gene [20] with a length of 2151 base pairs (bp), which was confirmed by amplifying the gene from the cDNA (Figure 1A). According to the sequence obtained, it was observed that 1362 bp correspond to the *g6pd* gene, and at its 3′ end the nucleotide sequence (789 bp) that includes the *6pgl* gene continues. On the other hand, it has been reported that in other protozoa the *6pgl* gene is located at the 5′ end of the *g6pd* sequence as occurs in *Theileria parva*, *Theileria annulata*, *Toxoplasma gondii*, *Neuspora caninun*, *Babesia bovis*, *Eimeria tenella*, and different *Plasmodium* species [19], which was confirmed by molecular and biochemical studies on *Plasmodium falciparum* [21,22]. Morales-Luna et al. [23] showed the fusion of *6pgl* with the *g6pd* gene also occurs in the protozoan parasite *Giardia lamblia* (*G. lamblia*), although it is located at the 3′ end, similar to the results deposited for the genome [20] and found in our results for *T. vaginalis*. An assumption mentioned by some authors is that the fusion between these genes may be due to a possible horizontal transfer [19,24] from bacteria, since these genes are normally found in the same operon in such organisms [25]. However, a primary difference between the G6PD fused with 6PGL in *T. vaginalis* and *G. lamblia* is that the encoding proteins vary in the number of their amino acids (aa), containing 716 and 742 aa, respectively.

Next, the fragment (2151 bp) obtained was cloned into a pJET vector to obtain and verify the sequence, which was subsequently translated into a protein (Supplementary Materials Figure S2), and showed 100% similarity to the sequence of *T. vaginalis* G3 with access number XP_001583274.1 when performing a blast nucleotide (BLASTn) analysis. As a result that TvG6PD::6PGL is similar to *G. lamblia*, which contains the 6PGL protein at its fused C-terminal end, we decided to compare the sequences of both protozoa by sequence alignment (Figure 1B) with three sequences of *T. vaginalis* and four of *G. lamblia*, which were obtained from the NCBI database. When analyzing these sequences, only a 30% similarity was found between both parasites, which is clearly seen in the alignment of the amino acids (Figure 1B). However, during the comparison of these sequences, certain conserved amino acids were also identified as previously reported for G6PD from *G. lamblia* (Figure 1A, blue boxes). The first fragment characteristic of G6PDs was found near the N-terminal end of the *T. vaginalis* protein, which corresponds to the amino acid 19-GxxGDLx-26 (the amino acid number corresponding to the G6PD region sequence from the TvG6PD::6PGL protein of *T. vaginalis*) and has been linked to coenzyme NADP^+^ binding [26]. Another conserved fragment was 163-EKPxG-167 (the amino acid number corresponding to the G6PD region sequence from the TvG6PD::6PGL protein of *T. vaginalis*), where a proline (P165) is present and which also participates in the correct positioning of the substrates (G6P) and the NADP^+^ coenzyme during the enzymatic reaction [26]. Lastly, the conserved fragment 191-RIDHYLAKE-199 region was also identified in *T. vaginalis*, which contains several amino acids, such as Lysine and Histidine, which are directly related to substrate binding and catalysis, as previously reported in *Leuconostoc mesenteroides* [27] and *Homo sapiens* [28,29]. Interestingly, when these regions of *Trichomonas* and *Giardia* were compared to human G6PD [26], there was no observable variation between them, since the consensus was very similar between the three regions analyzed (Figure 1C).

### 2.2. Homology Modeling of TvG6PD::6PGL

The annotated sequence of TvG6PD::6PGL retrieved from Trichdb [20] was used to perform a BLAST protein analysis against the Protein Data Bank (PDB), which found a target database of 3D structures. The TvG6PD::6PGL amino acid sequence has the best score and the highest similarity (37.5%) with the G6PD of *Homo sapiens* (PDB entry 2BH9) and 36.07% with the 6PGL from *Mycolicibacterium smegmatis* MC2 155 (PDB entry 3OC6).

**Figure 2:**
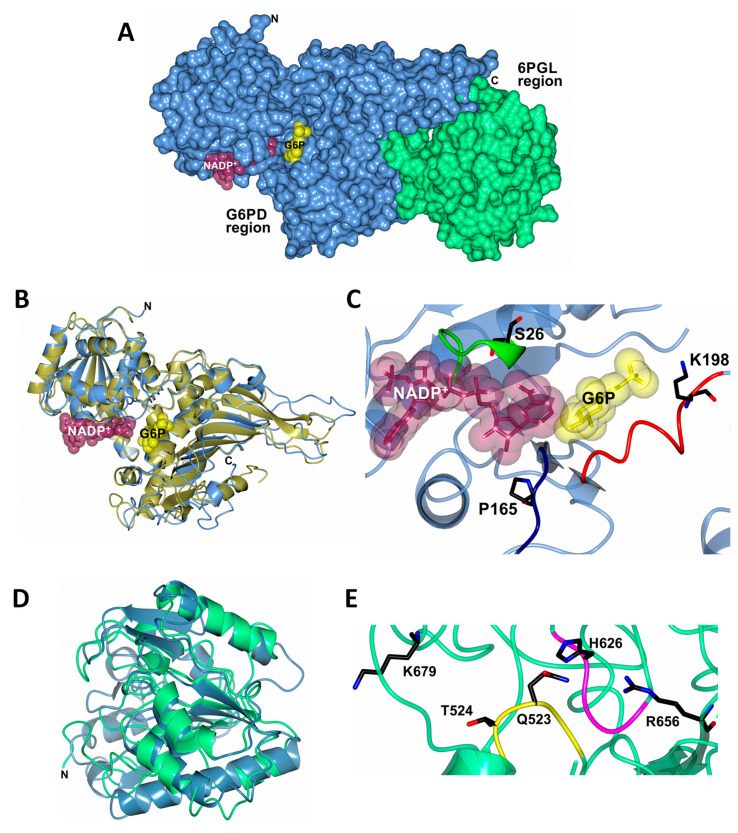
The homology model of the full-length TvG6PD::6PGL enzyme from *T. vaginalis*, (**A**) showing the N-terminal G6PD region (residues 1–483, cornflower blue) and the C-terminal 6PGL region (residues 484–712, spring green). The catalytic nicotinamide adenine dinucleotide phosphate (NADP^+^) and glucose-6-phosphate (G6P) substrate (Protein Data Bank (PDB) entries 2BHL and 2BH9) are drawn as dark purple and yellow molecular surface representations, respectively. (**B**) Structural superposition of the human G6PD crystal structure (PDB entry 2BH9, gold) with the N-terminal G6PD region of the TvG6PD::6PGL model (cornflower blue). (**C**) The G6PD active site showed the conserved sequences 20-GxxGDLS-26 (green), 163-EKPxG-167 (dark blue), and 191-RIDHYLGKE–199 (red). Representative residues (S26, P165, and K198) of these conserved sequences are shown as black cylinders. (**D**) Structural superposition of the 6PGL crystal structure from *Mycolicibacterium smegmatis* MC2 155 (PDB entry 3OC6, steel blue) with the C-terminal 6PGL region of the TvG6PD::6PGL model (spring green). (**E**) The 6PGL active site showed the conserved sequences 521-GGQTP-525 (yellow) and 625-GHTAS-629 (magenta). Representative residues (Q523 T524, and H626) of these conserved sequences, as well as R656 and K679 residues, are shown as black cylinders.

The model of the TvG6PD::6PGL protein was obtained on the Phyre2 (Protein Homology/analogY Recognition Engine V 2.0) server [30] (Supplementary Materials TvG6PD-6PGL_Phyre2.pdb). Furthermore, structural superposition of the TvG6PD::6PGL model before (ice blue cartoon) and after (gold cartoon) energy minimization using the YASARA force field was performed. As expected, structural changes in some unstructured loops, as well as some side-chains, were observed after energy minimization (Supplementary Materials Figure S3). The entire 3D TvG6PD::6PGL of the *T. vaginalis* model (Figure 2A) (Supplementary Materials TvG6PD-6PGL_Minimized.pdb) showed a total of 34 α-helices and 21 β-strands (Figure S4), and we did not find an extended α−helix (residues from 464 to 491) between the G6PD region and 6PGL region, as was observed previously in the model of G6PD::6PGL protein from *G. lamblia* [23].

Furthermore, a structural superposition of the G6PD crystal structure from human G6PD (PDB entry 2BH9) versus the TvG6PD::6PGL model from *T. vaginalis* (residues 1–483), showed a calculated r.m.s.d of 1.6 Å after superimposing 390 C^α^ (Figure 2B). We observed that the N-terminal of the TvG6PD::6PGL model (amino acids 1–198) contains the β-α-β Rossmann type folding domain, where the binding sites of β-d-glucose-6-phosphate (G6P) and NADP^+^ are located (Figure 2C). This agrees with the results previously reported for the human G6PD enzyme (PDB entry 2BH9) [26] and G6PD from *L. mesenteroides* (PDB entry 1H9A) [27], as well as for other G6PDs from different organisms. As shown in the alignment (Figure 1B,C), three conserved fragments of the G6PD region were identified and are located in the TvG6PD::6PGL model as follows. The first fragment sequences the amino acids 20-GxxGDLx-26 (the aa number corresponding to the G6PD region sequence from the TvG6PD::6PGL protein of *T. vaginalis*). This fragment, previously reported in human G6PD, is involved in binding the catalytic NADP^+^ coenzyme [26]. The second conserved fragment, 163-EKPxG-167 (the amino acid number corresponding to the G6PD region sequence from the TvG6PD::6PGL protein of *T. vaginalis*), contains proline 165 (P165), which is related to the correct positioning of the substrate (G6P) and coenzyme (NADP^+^) during enzymatic reactions [26]. The third fragment, 191-RIDHYLGKE-199 (the amino acid number corresponding to the G6PD region sequence from the TvG6PD::6PGL protein of *T. vaginalis*), contains lysine residue 198 (K198), is involved in substrate binding and catalysis (Figure 2C) [26].

Moreover, a structural superposition of the 6PGL crystal structure from *Mycolicibacterium smegmatis* MC2 155 (PDB entry 3OC6) compared to the 6PGL region from the *T. vaginalis* model (residues 484–713) showed a calculated r.m.s.d of 2.1 Å after superimposing 166 C^α^ (Figure 2D). We observed that although the number of amino acids in 6PGL varies from organism to organism, the Tv6PGL region also contains all the catalytic amino acids involved in the catalysis of 6-phosphoglucono-δ-lactone to gluconate (Figure 2E), which was confirmed by the multiple sequence alignment.

The first fragment corresponds to the amino acids 521-GGQTP-525 (the amino acid number corresponding to the 6PGL region sequence from the TvG6PD::6PGL protein of *T. vaginalis*), which contain the Q523 and T524 involved in the active site. Furthermore, we observed a fragment 625-GHTAS-629 that contains the H626 participle present in the enzymatic reaction of 6-phosphoglucono-δ-lactone. In addition, we found the residues R656 and K679, which are involved in the binding of 6-phosphoglucono-δ-lactone. Although we did not measure 6PGL activity in the fused TvG6PD::6PGL protein of *T. vaginalis*, the Tv6PGL region contains all the catalytic amino acids involved in substrate binding and catalysis (Figure 2E) [26].

### 2.3. Expression and Purification of the TvG6PD::6PGL Protein

**Figure 3:**
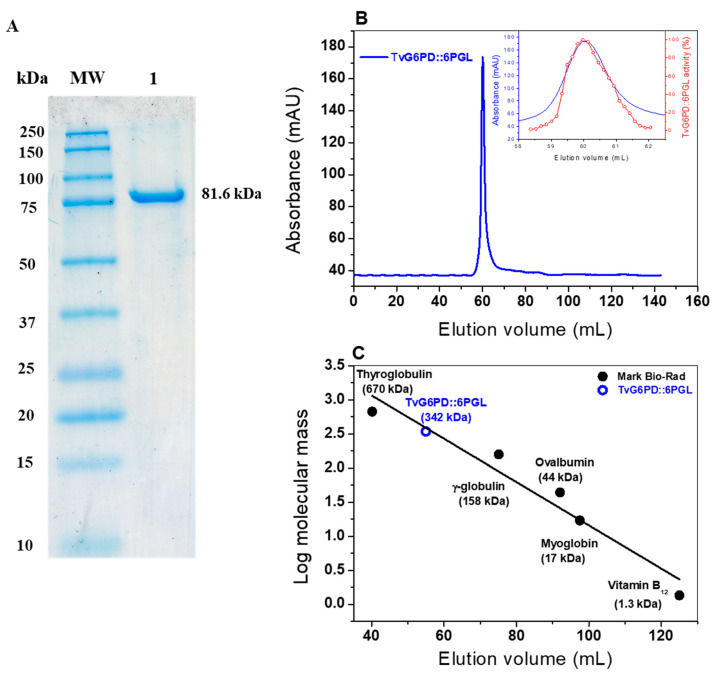
Purification and native status of the TvG6PD::6PGL protein. (**A**) Polyacrylamide gel electrophoresis SDS-PAGE (12%) of the recombinant TvG6PD::6PGL protein. M: Protein molecular weight (MW) marker precision plus protein kaleidoscope standards from Bio-Rad (Bio-Rad, Hercules, CA, USA). Lane 1. TvG6PD::6PGL purified protein (10 µg). (**B**). Size exclusion chromatography of TvG6PD::6PGL. Inset: Blue line indicates the TvG6PD::6PGL purified protein, and the red line represents the TvG6PD::6PGL activity. (**C**) Calibration curve showing the elution volumes versus the log of the molecular weight (MW) of the Bio-Rad gel filtration standard (●). MW of the TvG6PD::6PGL protein (●).

*Escherichia coli* BL21(DE3)Δ*zwf*::kan^r^ cells were used to produce the recombinant protein. The G6PD::6PGL protein was expressed with 0.3 mM of isopropyl-β-D-thiogalactopyranoside (IPTG) and purified by a Ni Sepharose high performance affinity column (GE Healthcare, Chicago, IL, USA). The protein was analyzed by SDS-PAGE gel, showing a single band with an apparent molecular weight (MW) of 81.6 kDa (Figure 3A). This result is consistent with the molecular weight (MW) of 81,692 Da and the theoretical isoelectric point (pI) of 6.62, which was determined with the ProtParam tool from the Expasy online program [31]. This result is also similar to the reported molecular mass of the fused G6PD::6PGL protein from *G*. *lamblia* [23], although it differs from the PbGlc6PD from *Plasmodium berghei* (110 kDa) [21] and the PfGlc6PD (107 kDa) from *P. falciparum* [22]. Supplementary Table S1 provides a summary of the purification process, showing the amount of protein obtained (12.4 mg), its specific activity (108.6 µmol min^−1^ mg^−1^), and the yield of purified protein (47.5%).

### 2.4. Functional Analysis of the TvG6PD::6PGL Protein

#### 2.4.1. Native Status of the TvG6PD::6PGL Protein

The oligomeric state of the protein in the solution was determined by size exclusion chromatography. As shown in Figure 3B, a single peak with an elution volume of 59.9 mL featuring G6PD activity was observed in the chromatogram (Figure 3B; Inset). To determine the native status of the protein, we produced a calibration curve plot and found that the protein mass corresponds to the native tetramer (Figure 3C), with an MW of 342 kDa, which is in accordance with the MW expected from the amino acid sequence (81.6 kDa × 4 ≈ 326 kDa). Further, no monomer MW, dimer MW, or larger aggregates were observed in the fast protein liquid chromatography (FPLC) chromatogram. However, this native status determined for the fused TvG6PD::6PGL differed with that previously reported for the fused G6PD::6PGL from *G. lamblia*, where active native dimers were observed. These differences in native status in the G6PD::6PGL protein from *T. vaginalis* and *G. lamblia* could be mainly because both enzymes are different in number (716 aa in G6PD::6PGL from *T. vaginalis*, and 742 aa in G6PD::6PGL from *G. lamblia*), and composition of amino acids, as was previously observed in the alignment of the amino acid sequences of *T. vaginalis* and *G. lamblia*, where only a 30% similarity was found between both parasites (Figure 1B). In addition, the native status determined for the G6PD::6PGL protein from *T. vaginalis* is in concordance with the previously reported G6PDs (*Brugia malayi* and *Pseudomonas aeruginosa*), for which a tetrameric structure was reported [32,33].

#### 2.4.2. Effect of Temperature and pH on TvG6PD::6PGL Activity

**Figure 4:**
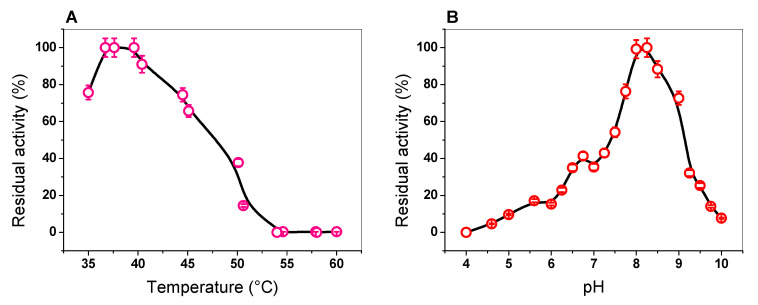
Effect of temperature and pH on the activity of the TvG6PD::6PGL protein. (**A**) The heat-inactivation profile of G6PD activity (pink circle). (**B**) Effect of pH on G6PD activity (red circle). The fused TvG6PD::6PGL protein was incubated at different temperatures (35–60 °C) for 20 min in 50 mM Tris buffer at pH 8.0. Error bars indicate the mean ± standard deviation of the triplicate values.

To determine the effect of temperature on G6PD activity, a temperature gradient (35–60 °C) was produced to measure changes in activity. A graph with the activities obtained at different temperatures is shown in Figure 4A. The greatest activities were observed at temperatures from 36.5 to 39.6 °C. G6PD activity dropped rapidly when the temperature exceeded 44 °C and almost all activity ceased above 55 °C. This loss of activity observed in the TvG6PD::6PGL protein is similar to that previously observed in *G. lamblia*, where the same loss of activity was observed as a function of temperature. In addition, from the plot, we observed that the TvG6PD::6PGL protein displayed a T_1/2_ (the temperature at which the enzyme loses 50% of its original activity) of 47 °C, which is in concordance with the previously reported value for the fused G6PD::6PGL protein from *G. lamblia*, where a T_1/2_ of 49 °C was obtained, thus reflecting the high stability of both enzymes’ active sites [23]. 

On the other hand, to determine the effect of pH on the G6PD activity of the recombinant TvG6PD::6PGL protein, we examined the activity at different pH values (between 4.0 and 10). According to the results (Figure 4B), the TvG6Pd::6PGL protein did not show the classical bell-shape observed for most enzymes and was similar to the previously reported results for the fused G6PD::6PGL protein from *G. lamblia*. We observed that at pH values from 4.0, no G6PD activity was present. However, at a pH value of 5.0, the TvG6Pd::6PGL protein showed 10% G6PD activity. Furthermore, we observed a small peak with a relative activity of 41% at pH 6.7, but at a pH value of 8.0, a peak of 100% activity was observed. Finally, at pH values from 8.2 to 10.0, residual activity of 60% and 7.6% was observed (Figure 4B). This result is different with that previously reported for the fused G6PD::6PGL protein from *G. lamblia*, where the enzyme reached a maximum value at pH 8.75. In this way, the optimal pH determined for TvG6PD::6PGL agrees with that of the other previously purified G6PDs, where pH values of 8.0 were found in G6PDs (in *Homo sapiens*, *B. malayi*, buffalo liver, camel liver, dog liver, *Taenia crassiceps*, *Trypanosoma cruzy*, *Aspergillus Oryzae*, *Thermotoga maritima*, *Pseudomonas aeruginosa*, and *E. coli* DH5α [32,33,34,35,36,37,38,39,40]. Considering these data, the rest of the functional assays for our TvG6PD::6PGL enzyme were performed at a pH of 8.0. 

#### 2.4.3. Kinetic Characterization of the TvG6PD::6PGL Enzyme

**Figure 5:**
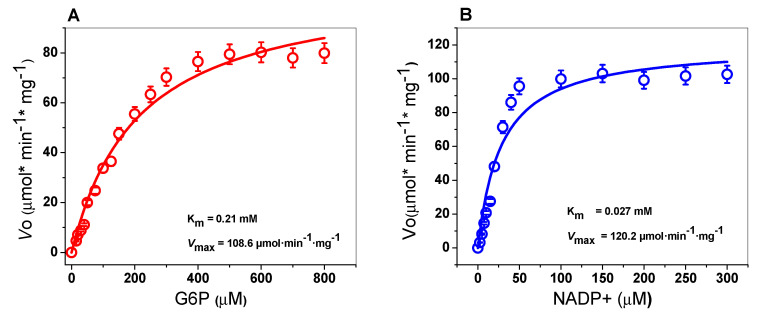
Michaelis–Menten plots for fused TvG6PD::6PGL with (A) G6P and (B) NADP^+^ as substrates. The data represent the mean ± SD from five independent experiments.

The steady-state kinetic parameters were determined spectrophotometrically by measuring the reduction of the NADP^+^ at 340 nm by varying one of the substrates (G6P or NADP^+^), while the second substrate (G6P or NADP^+^) remained fixed. The TvG6D::6PGL enzyme showed hyperbolic behavior for both substrates (G6P or NADP^+^) when measuring the G6PD activity (Figure 5). The apparent K*_m_* values for G6P and NADP^+^ were 0.21 and 0.027 mM, respectively (Table 1), with an apparent *V*_max_ of 108.6 µmol·min^−1^·mg^−1^. A comparison of the kinetic parameters of G6PDs of different microorganisms, and human G6PD is shown in Table 1. We observed that the K*_m_* value for NADP^+^ obtained in TvG6PD::6PGL protein is similar with the K*_m_* value of NADP^+^ on the G6PD from *T. cruzi* [40] and is higher than with the previously reported in G6PDs from *G. lamblia* [23], *P. falciparum* [22], *P. vivax* [41], and *Homo sapiens* [42]. With respect to the K*_m_* value for G6P determined in TvG6PD::6PGL protein from *T. vaginalis*, it is similar with the K*_m_* value reported for G6PD from *T. brucei* [43], and higher than the previously reported values in G6PDs from *G. lamblia* [23], *P. falciparum* [22], *P. vivax* [41], *T. cruzi* [40], and *Homo sapiens* [42], respectively. Moreover, as shown in Table 1, the fused TvG6PD::6PGL protein presented a high catalytic constant (*k_cat_*) value (147 s^−1^) with respect to the fused G6PD::6PGL proteins from parasites such as *G. lamblia* (31 s^−1^) [23], *P. falciparum* (8 s^−1^) [22], and G6PD from *T. cruzi* (53 s^−1^) [40]. However, the catalytic constant (*k_cat_*) value determined for the TvG6PD::6PGL protein was lower compared to human G6PD (233 s^−1^) (non-fused) [34] and non-fused G6PD from *G. diazotrophicus* (293,181 s^−1^) [44].

### 2.5. Evaluation of the Stability of the TvG6PD::6PGL Protein by a NADP^+^ Molecule

#### 2.5.1. Thermal Inactivation Analysis

**Figure 6:**
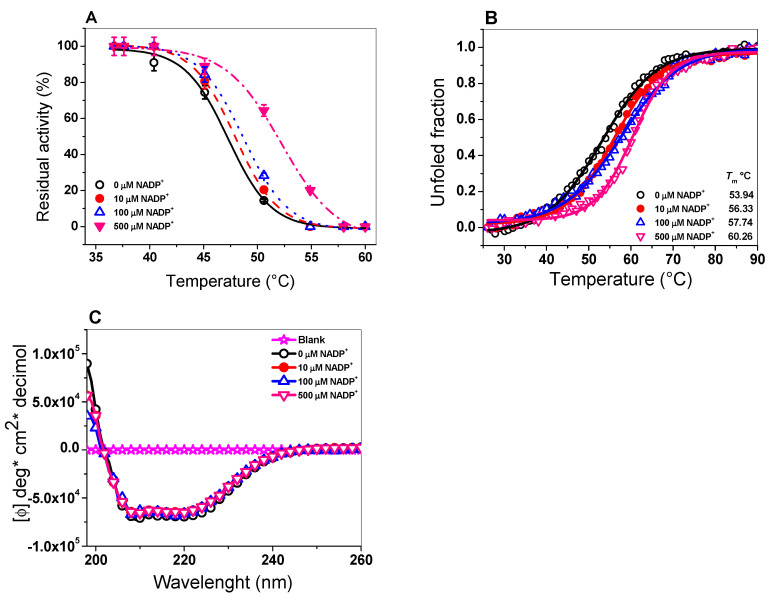
Evaluation of the protein stability of the TvG6PD::6PGL protein. (**A**) Thermal inactivation assays of the TvG6PD::6PGL protein. (**B**) Thermal stability of the Tv G6PD::6PGL protein, and (**C**) Far-UV circular dichroism (CD) spectra of the TvG6PD::6PGL protein. These experiments are representative of triplicate experiments.

It was previously reported that the human WT G6PD enzyme facilitates the secondary binding of the NADP^+^ molecule known as the structural NADP^+^ binding site, which is crucial for the long-term stability of the enzyme. For this reason, we evaluated the effect of the NADP^+^ molecule on the protein stability of the TvG6PD::6PGL protein via thermal inactivation analysis. As seen in Figure 6A, when the enzyme was incubated with 10 µM of NADP^+^, no protective effect was observed on residual activity, since the value of T_50_ (the temperature at which the enzyme loses 50% of its initial activity) was 47.06 °C and remained similar without NADP^+^ (47.76 °C). However, when the TvG6PD::6PGL protein was incubated with a higher concentration of NADP^+^ (500 µM), its residual activity was increased by 6 °C (a T_50_ of 51.93 °C) without the NADP^+^ molecule. This result is similar to that for the fused G6PD::6PGL protein from *G. lamblia*, whose T_50_ increased by 6 °C (from 49.3 to 57.4 °C) when it was incubated in the presence of 500 µM of NADP^+^ [45]. These results indicate that the protein became more thermoresistant than without NADP^+^, showing a shift of 6 °C in the presence of NADP^+^. This protective effect could indicate the presence of a structural NADP^+^ binding site in the TvG6PD::6PGL protein.

#### 2.5.2. Analysis of the Thermal Stability of the TvG6PD::6PGL Protein

Previously it was demonstrated that the NADP^+^ molecule exerts a protective effect on recombinant human G6PD and the fused G6PD::6PGL from *G. lamblia*. In thermal inactivation assays, a protective effect on the residual activity of the TvG6PD::6PGL protein was observed when the protein was incubated with different concentrations of NADP^+^ (from 10 to 500 µM) after being incubated at different temperatures. We performed an assay to analyze the structural stability of the TvG6PD::6PGL protein with the same concentration of NADP^+^ across circular dichroism (CD) following changes in the α–helices at 222 nm. Figure 6B shows that the *T_m_* value of the TvG6PD::6PGL without NADP^+^ was 53.9 °C. Moreover, an increase in the *T_m_* values was observed when the concentration of NADP^+^ was increased (60.2 °C at 500 µM). This result indicates that the protein became more resistant to unfolding due to temperature than without the NADP^+^ molecule, where a shift of 6.3 °C in the *T*m value was observed in the presence of NADP^+^ (500 μM) than without NADP^+^. This effect suggests that the TvG6PD::6PGL protein likely has a structural NADP^+^ coenzyme binding site, as seen in the thermal inactivation assay, which confers global stability to the protein. However, TvG6PD::6PGL is less stable at 5.5 °C compared to human G6PD, where a Tm value of 59.5 °C was reported in the absence of NADP^+^ [46]; this is also 3 °C less than the *T_m_* value for the fused G6PD::6PGL from *G. lamblia* (*T_m_* = 57 °C) [23]. The differences between these *T_m_* values are likely due to the absence of conserved amino acids to bind structural NADP^+^ in the TvG6PD::6PGL protein, as mentioned in the thermal inactivation assay.

#### 2.5.3. Circular Dichroism (CD) of the TvG6PD::6PGL Protein

To determine whether the changes observed in the thermal stability assay of the TvG6PD::6PGL protein in the presence of the NADP^+^ molecule were due to alterations in the secondary structure of the protein, we evaluated the secondary structure of the recombinant TvG6PD::6PGL protein in the presence of the same NADP^+^ concentrations by CD. The ultra-violet circular dichroism (UV–CD) spectra of the TvG6PD::6PGL protein presented wave emissions in the molar ellipticity at 208 and 222 nm (Figure 6C). The UV–CD spectra did not show changes in the secondary structure of the protein (α–helices and β–sheets), when the protein was incubated with different NADP^+^ concentrations, indicating that the NADP^+^ molecule did not alter the folding of the protein at the level of the secondary structure and that the protective effect observed in the thermal inactivation and thermal stability trials could be due to the presence of the structural NADP^+^ binding site in the TvG6PD::6PGL protein. This result is in agreement with that previously reported for the fused G6PD::6PGL from *G. lamblia* [45], where no alterations in molar ellipticity, either in the absence or presence of the NADP^+^ molecule, were observed. This means that the shift in thermal stability and the thermal stability observed for the TvG6PD::6PGL protein in the presence of the NADP^+^ molecule are due to a protective effect, as also reported for the human G6PD [44] and fused G6PD::6PGL from *G. lamblia* [45].

#### 2.5.4. Susceptibility of the TvG6PD::6PGL Protein to Trypsin Digestion

**Figure 7:**
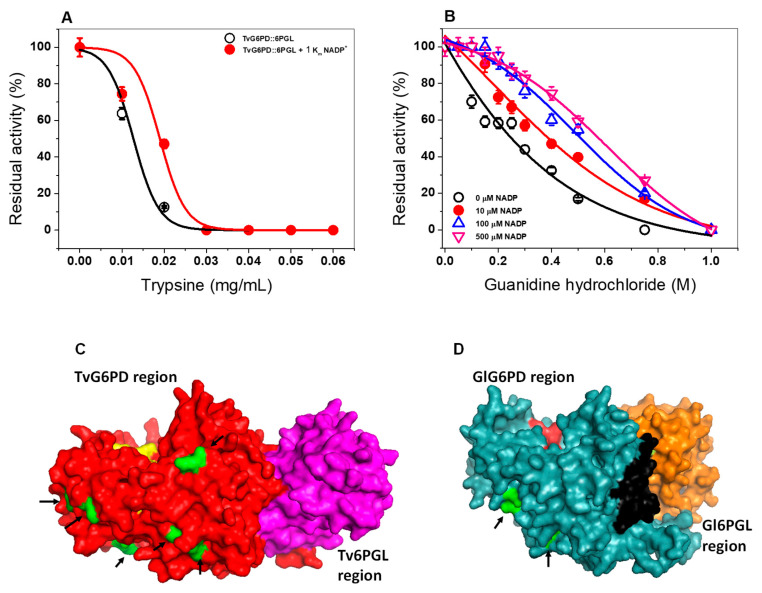
Evaluation of the stability of the TvG6PD::6PGL protein by trypsin digestion and guanidine hydrochloride (Gdn-HCl). (**A**) The trypsin enzymatic digestion of TvG6PD::6PGL in the presence of 1 K_m_ of NADP^+^ and different concentrations of trypsin. (**B**) Activity of the TvG6PD::6PGL protein in the presence of Gdn-HCl and three NADP^+^ concentrations. For both assays, the protein was incubated for 2 h at 37 °C, and the residual activity was measured. (**C**) A homology model of the full-length TvG6PD::6PGL protein and (**D**) G6PD::6PGL protein model from *G. lamblia*, showing the regions (in green) susceptible to digestion with trypsin (also indicated with arrows in both models).

Another approach to analyzing the effect of the NADP^+^ molecule on the stability of the TvG6PD::6PGL protein was through susceptibility to trypsin digestion. Therefore, we assess the impact of trypsin in presence of 1 K*_m_* NADP^+^ concentrations (27 µM). As the concentration of trypsin was increased (0 to 0.06 mg/mL), the activity of the TvG6PD::6PGL protein decreased until no activity was detected (Figure 7A). When the TvG6PD::6PGL protein was incubated with 27 µM of NADP^+^, a slight protective effect in its resistance to protease digestion was observed. This result is in concordance with that previously observed for the fused G6PD::6PGL protein from *G. lamblia* [45], and human G6PD where a protective effect was observed when the protein was incubated with NADP^+^ and the proteins became more resistant to protease digestion compared to without NADP^+^ [45,47]. However, the TvG6PD::6PGL protein was more susceptible to trypsin protease degradation compared to the fused G6PD::6PGL protein from *G. lamblia* and the G6PD proteins from *G. diazotrophicus* and *H. sapiens,* where 0.4 mg/mL of trypsin was necessary to abolish 50% of the initial activity [23,43,45]. The TvG6PD::6PGL protein was thus more susceptible to trypsin digestion than the fused G6PD::6PGL protein from *G. lamblia* and human G6PD because the TvG6PD::6PGL protein features eight types of cleavage-specific trypsin digestion, as shown in Figure 7C, while the fused G6PD::6PGL from *G. lamblia* and human G6PD proteins contains only two specific trypsin cut sites (Figure 7D).

#### 2.5.5. Susceptibility of TvG6PD::6PGL to Gdn-HCl

The effect of the NADP^+^ molecule on the stability of the recombinant enzyme TvG6PD::6PGL was evaluated in the presence of chaotropic agents, such as guanidine hydrochloride (Gdn-HCl) (between 0 to 1 M) and different NADP^+^ concentrations (10, 100, and 500 μM). As seen in Figure 7B, when incubated with a chaotropic agent, the enzyme gradually loses its activity until reaching a total loss, which is observed at a concentration of 1.0 M Gdn-HCl. We determined that the Gdn-HCl_1/2_ value (the Gdn-HCl concentration at which the protein loses 50% of their original activity after 2 h at 37 °C) was 0.5 M for Gdn-HCl. This Gdn-HCl_1/2_ value reveals that the TvG6PD::6PGL protein is more resistant to the denaturation exerted by chaotropic agents related to other G6PDs, such as those of *H. sapiens* (0.3–0.45 M) [47] and even those of *G. lamblia* [23]. In the presence of 500 µM of NADP^+^, the progressive loss of residual activity was Gdn-HCl concentration-dependent since, at low concentrations of Gdn-HCl (0 to 0.2 M), the enzymatic activity of the TvG6PD::6PGL enzyme did not change, but at concentrations ranging from 0.3 to 0.70 M, the activity decreased gradually until it become imperceptible (down to 1 M of Gnd-HCl). On the other hand, when the TvG6PD::6PGL protein was incubated with NADP^+^ concentrations (10 µM, 100 µM, and 500 µM) and incubated again with Gnd-HCl, the Gdn-HCl_1/2_ values determined for 10 µM, 100 µM, and 500 µM were 0.5 M, 0.58, and 0.64 M, respectively. These results indicate that the recombinant fused TvG6PD::6PGL protein was again protected by the NADP^+^ molecule, which could favor binding the molecule at the structural site of the enzyme, as reflected by the greater stability of the protein. These data are in accordance with the results formerly obtained in the thermostability ramp in the presence of NADP^+^, where a shift in the *T_50_* values was observed in the presence of 100 µM and 500 µM of the NADP^+^ molecule. These results for the fused TvG6P*D*::6PGL protein are in agreement with those previously reported for another G6PD::6PGL protein from *G*. *lamblia*, a basal eukaryote, where the existence of a structural NADP^+^ site was confirmed via biochemical assays [45]. These experimental results indicate the presence of a structural NADP^+^ binding site in the TvG6PD::6PGL protein, as it was possible to observe the protective effect caused by the NADP^+^ molecule.

### 2.6. Structural and Spectroscopic Characterization

#### Determination of the *Kd* value of Tv G6PD::6PGL from *T. vaginalis*

**Figure 8:**
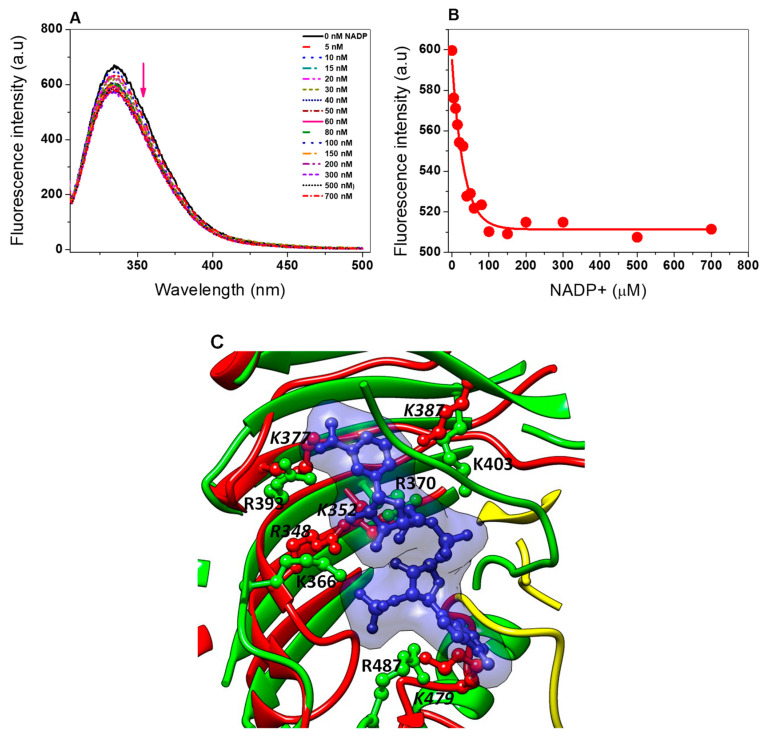
Binding of the dinucleotide structural cofactors to TvG6PD::6PGL. (**A**) The dependent fluorescence signal intensity was measured in 50 mM phosphate buffer, pH 7.4. Changes in fluorescence were normalized to the value of the initial fluorescence of the protein. The arrow indicates the decreased in intrinsic fluorescence. (**B**) Data for the total fluorescence intensity were fitted to non-linear regression calculations. The experiments were performed in triplicate; the standard errors were less than 5%. (**C**) Zoom of structural superposition of the human G6PD crystal structure (PDB entry 2BH9, green cartoon) with the TvG6PD::6PGL model (red cartoon) in the probably structural NADP^+^ binding site in TvG6PD::6PGL from *T. vaginalis*. Numbering of residues in the human and *T. vaginalis* enzymes are given in bold and italics bold, respectively.

Previously, Morales-Luna et al. [45] suggested the presence of a structural NADP^+^ binding site in the fused G6PD::6PGL from *G. lamblia*, as demonstrated in the human G6PD, where one molecule of structural NADP^+^ by subunit is present in the crystal structure of the variant G6PD Canton [48], which confers structural stability and is necessary for dimerization of the protein [37]. As a result that we observed a protective effect of the NADP^+^ molecule in the TvG6PD::6PGL protein for thermal inactivation, thermal stability, susceptibility to proteolysis, and susceptibility to Gdn-HCl assays, we determined the ligand dissociation constant (*K_d_*) of the structural NADP^+^ in the TvG6PD::6PGL protein. As seen in Figure 8, as the protein was titrated with increasing NADP^+^ concentrations, the maximum fluorescence intensity of the proteins decreased. The stripped TvG6PD::6PGL enzyme gave a maximal fluorescence emission spectrum of 528 arbitrary units (a.u), with a maximum peak at 344 nm (Figure 8A). However, at high NADP^+^ concentrations (from 200 to 500 µM), the fluorescence intensity was partially quenched until reaching 404 arbitrary units (a.u) with a maximum peak at 344 nm. To determine the dissociation constant (*K*_d_) value, we plotted the NADP^+^ concentration versus the maximum fluorescence emissions, and the data were fitted to non-linear regression calculations with the equation for a single binding constant [45] after correcting the inner filter effect using the Origin program. The *K*_d_ value calculated from the concentration dependence of the decrease in fluorescence was 31 nM for the TvG6PD::6PGL protein from *T. vaginalis*, which showed an 870-fold higher affinity compared to catalytic NADP^+^ (K_m_ = 27 μM) (Figure 8B). This result agrees with that previously reported by Wang et al. [49] for human G6PD, where the *K*_d_ value was determined to be 37 nM, and for the fused G6PD::6PGL protein from *G. lamblia*, where Morales-Luna et al. [45] measured a *K*_d_ value of 63 nM (which were 200-fold and 219-fold lower, respectively, than catalytic NADP^+^). The dissociation constant (*Kd*) value for the NADP^+^ molecule in the fused G6PD::6PGL protein from *T. vaginalis* indicates that this protein likely contains a structural NADP^+^ binding site, which confirms the results previously observed in the stability trials, where a protective effect in the protein was observed, suggesting that the G6PD::6PGL enzyme from *T. vaginalis* has the ability to bind to structural NADP^+^, although the TvG6PD::6PGL does not contain all the amino acids required to bind the structural NADP^+^ molecule, as occurs in the human G6PD enzyme, where among the 11 amino acid residues that participate in the union of structural NADP^+^ in human G6PD (Lys238, Lys366, Arg370, Arg393, Tyr401, Lys403, Asp421, Thr423, Arg487, Tyr503, and Trp509; amino acid number corresponding to human G6PD), only one residue is conserved in TvG6PD::6PGL protein (HuG6PD K403 → TvG6PD::6PGL K387) with human G6PD as seen through a structural alignment (Figure 8C). In addition, we observed that other four residues (HuG6PD K366 → TvG6PD::6PGL R348, HuG6PD R370 → TvG6PD::6PGL K352, HuG6PD R393 → TvG6PD::6PGL K377, and HuG6PD R487 → TvG6PD::6PGL K479) could be involved in the binding of structural NADP^+^ because the basic residue K366, R370, R393, and R487 in human G6PD has been replaced by another basic residue R348, K352, K377, and K479 in TvG6PD::6PGL protein (Figure 8C); which does not cause drastic changes in their physicochemical properties and we consider that this may be sufficient for the NADP^+^ molecule to bind to the protein. These results are in concordance with the previously reported by Morales-Luna et al. [23] were through a structural alignment between HuG6PD and fused G6PD::6PGL protein from *G. lamblia*, they observed that four residues were spatially placed in the same position as that of the corresponding human structural NADP^+^ binding site (PDB entry: 2BH9) [26]. 

**Figure 9:**
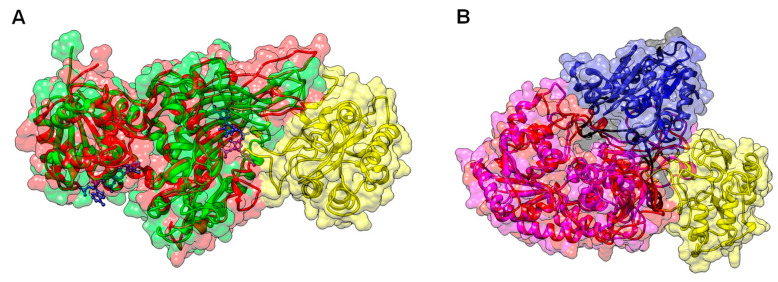
Structural alignment of the minimized model of TvG6PD::6PGL. (**A**) Structural alignment of the human G6PD enzyme (PDB entry 2BH9; green cartoon) and the minimized model of the TvG6PD::6PGL from *T. vaginalis* (G6PD domain, red cartoon; 6PGL domain, yellow cartoon). The catalytic NADP^+^ and the structural NADP^+^ from human G6PD are shown as blue sticks. (**B**) Structural alignment of the minimized model of TvG6PD::6PGL from *T. vaginalis* (G6PD domain, red cartoon; 6PGL domain, yellow cartoon) and the minimized model of the fused G6PD from *G. lamblia* (the G6PD domain is represented in magenta cartoon, the 6PGL domain is represented in blue cartoon, and the dark-colored loop is located between the G6PD and 6PGL interfaces).

In addition, in the TvG6PD::6PGL model, we observed the presence of the β + α domain that forms the dimer interface and contains a large antiparallel sheet in the C-terminal G6PD region, which has been proposed to be the site of binding for a second additional NADP^+^ (structural) that confers stability to the G6PD protein in higher organisms (Figure 9A) [26,48]. According to our results, the TvG6PD::6PGL protein has the ability to bind to the second structural NADP^+^ because the enzyme becomes more resistant to temperature both in its active site (thermal inactivation assay) and due to the global stability of the protein (thermal stability assay). We also observed the protective effect of the NADP^+^ molecule in the presence of trypsin and Gdn-HCl, which is consistent with the idea that this site is only present in higher organisms and parasites, as observed in *G. lamblia* [45].

Finally, when comparing the 3D structural model of the G6PD::6PGL from *T. vaginalis* to the model obtained from *G*. *lamblia*, we observed that although both proteins are fused important differences between them are observed in the structural superposition of TvG6PD::6PGL model from *T. vaginalis* versus the fused G6PD::6PGL model from *G. lamblia*, where showed a calculated r.m.s.d of 1.63 Å after superimposing 403 C^α^ (Figure 9B). In addition, when an alignment of amino acid sequences of *T. vaginalis* and *G. lamblia* was performed, only a 30% similarity was found between both sequences from parasites, as previously observed in the Figure 1B. These structural differences compared to human G6PD make both fused G6PD::6PGL proteins from parasites like *T. vaginalis* and *G*. *lamblia* ideal targets for drug development, as the same approach has been used successfully for other parasites, such as *P*. *falciparum* and *Trypanosoma cruzy* [18,22]. In addition, the obtained TvG6PD::6PGL 3D structural model could help in future studies to determine the binding site of specific drugs through docking analyses and to assist in the search for molecules that can bind to amino acids of certain regions of the proteins, as the use of small-molecule inhibitors that have been previously reported by Preuss et al. [50] in the fused glucose-6-phosphate dehydrogenase 6-phosphogluconolactonase from *Plasmodium facilparum*. These molecules could be used as scaffolds to develop new molecules that inhibit the fused TvG6PD::6PGL protein from *Trichomonas vaginalis* but that not inhibit at the human G6PD enzyme; and which could be use as target to block the PPP metabolic pathway of the parasite.

## 3. Materials and Methods 

### 3.1. Culture, RNA Extraction, and cDNA

The trophozoites of *T. vaginalis* were isolated from male urine and grown in a Diamond trypticase, yeast extract and maltose medium (TYM medium) pH 5.0 [51] supplemented with heat-inactivated serum from a horse (10%), and antibiotics (cephalothin, ampicillin, and amphotericin, 10, 10, and 5 mg/mL, respectively) at 37 °C. The trophozoites were recovered by centrifugation (3700 rpm for cultures at an exponential phase for 10 min) and washed with phosphate buffered saline (PBS) (1.5 mM KH_2_PO_4_, 14 mM Na_2_HPO_4_, 2.8 mM KCl, 137 mM NaCl, pH 7.0) [52]. RNA extraction was performed with Trizol (Invitrogen, Carlsbad, CA, USA) following the procedure described by the manufacturer and as reported by Morales-Luna et al. [23]. The RNA concentration was measured on Nanodrop ND-1000 equipment. Then, 1 µg of RNA was treated with DNAseI (Thermo scientific, Waltham, MA, USA), and 200 ng of RNA was used to obtain cDNA using the oligo(dT)_18_ primer and the *Revertaid* Reverse Transcriptase enzyme (Thermo scientific, Waltham, MA, USA).

### 3.2. Amplification and Cloning of the g6pd::6pgl Gene

The *Tvg6pd::6pgl* gene was obtained by endpoint PCR. The primers used for amplification were G6PD forward (GCGGCATATGACTTCTACCTTTTACG) and G6PD reverse (AATTGGATCCTTATACGACATGGAACC), which also included the sequences for restriction enzymes *Nde*I and *Bam*HI (indicated in bold), respectively. The PCR reaction mix was performed as follow: cDNA (100 ng) as a template, primer (200 ng), deoxynucleotides (dNTPs) (1 mM), MgCl2 (2 mM), 1× high fidelity (HF) buffer, and with the Phusion enzyme^®^ High Fidelity DNA polymerase (1 U) (Thermo scientific, Waltham, MA, USA). Amplification conditions were similar to previously reported by Morales-Luna et al. [23].

The amplicons obtained were visualized on agarose gels (1%) and stained with GelRed (Nucleic Acid Gel, Biotium, USA). Next, the fragment of interest was purified from the gels and after cloned into a maintenance vector pJET1.2 (Thermo scientific, Waltham, MA, USA) in order to obtain the sequence of the *g6pd::6pgl* gene. Finally, the gene was subcloned into the pET3a-HisTEV expression vector to obtain the plasmid named pTvG6PD::6PGL, and used to express the recombinant protein in the *E. coli* BL21(DE3)∆zwf::kan^r^ strain. All transforming colonies were selected from the plates with a Luria Bertani (LB) medium containing ampicillin (100 μg/mL).

### 3.3. Bioinformatic and Alignment

The *g6pd::6pgl* gene sequence with access number TVAV_414060 was obtained from the TrichDB database (https://trichdb.org/trichdb/) [20], which was used to design the primers. The ProtParam program was used to obtain the theoretical molecular mass and isoelectric point (pI) of TvG6PD::6PGL. For alignment, the nucleotide sequence obtained from the gene amplified by PCR was translated into protein via the translation software, ExPASy (https://web.expasy.org/translate/), and the sequence was analyzed in a protein blast from the NCBI [53]. Multiple sequence alignments were done via ClustalW with the online program from the EMBL-EBI.

### 3.4. Homology Modeling and Comparison of TvG6PD::6PGL

The sequence of the TvG6PD::6PGL protein that comes from the translation of the gene obtained by PCR and which shows 100% identity with sequence EAY09755.1 deposited in the Gene Bank database (NCBI), was used to perform the protein modeling. The homology model of the TvG6PD::6PGL protein was obtained using the Phyre2 (Protein Homology/analogY Recognition Engine V 2.0) server [30] (Supplementary Materials TvG6PD-6PGL_Phyre2.pdb), employing a profile-profile alignment algorithm against the fold library of *Phyre2*. Hence, the full-length 3D structure with 98% of the residues modeled at >90% confidence [26], was built using multiple protein templates with known crystallographic structures. The model was subjected to energy minimization using *YASARA* force field (Supplementary Materials TvG6PD-6PGL_Minimized.pdb), in order to correct wrong side-chain contacts and unsuitable torsion angles [54] and then validated using *MolProbity* to evaluate the model’s quality (energy and structure) at both the global and local levels [55]. A structural analysis was performed via manual inspection using Coot [56] and the PDBsum tool [57]. The graphical representations were made using the CCP4mg version 2.10.6 software [58].

### 3.5. Expression and Purification

The recombinant TvG6PD::6PGL protein was expressed in the *E. coli* BL21(DE3)pLysS strain containing the plasmid pTvG6PD::6PGL in LB medium (20 mL). Next, this medium was used to inoculate 2 L of the LB medium supplemented with 100 μg/mL ampicillin (Sigma Aldrich, St. Louis, MO, USA) and kanamycin (50 μg/mL). The bacterial culture was grown for 8 h at 37 °C and 160 rpm. Upon achieving a culture at optical density (OD 600 nm) of 0.8, the protein was induced with 0.3 mM of isopropyl β-D-thiogalactopyranoside (IPTG), the temperature was changed to 25 °C, and the culture was maintained for 8 h. Next, the cells expressing the (His 6×) TvG6PD::6PGL protein were collected by centrifugation at 4000× *g* over 20 min at 4 °C and resuspended in a lysis buffer (50 mM KH_2_PO_4_; 150 mM NaCl; 2 mM DTT; 5 µM NADP^+^; 10 % glycerol; 0.5 mM phenylmethylsulfonyl fluoride (PMSF); 1.4 mM β-mercaptoethanol; 50 mM imidazole; pH 7.35). Then, the cells were sonicated with 15 pulses of 45 s with rest intervals of 2 min. The lysate was centrifuged at 14,500× *g* for 30 min, and the clear supernatant obtained was labeled as the crude extract.

Then, the crude extract was incubated with a Ni Sepharose high performance column previously equilibrated with 10 bed-volumes of equilibrium buffer (50 mM KH_2_PO_4_; 150 mM NaCl; 5 µM NADP^+^, 2 mM DTT, and Glycerol 10%; pH 7.35) at 22 °C under stirring for 45 min and the column was washed with equilibrium buffer plus 50 mM imidazole. The protein was eluted with the same equilibrium buffer plus 250 mM imidazole [44], and the imidazole was removed from the TvG6PD::6PGL protein by consecutive dilutions using a microcon-10 kDa centrifugal filter unit (Millipore). The TvG6PD::6PGL protein was digested with the Tobacco Etch Virus Protease (TEVP) protease (previously purified) to remove additional amino acid residues corresponding to the (His)6-tag sequence located in the N-terminal region of the protein. The TvG6PD::6PGL protein was concentrated, and imidazole was removed from the sample by five consecutive dilutions using a Centricon-10 kDa centrifugal filter unit (Millipore, Burlington, MA, USA).

Protein purity was verified on 12% SDS-PAGE gels and that were stained with colloidal Coomassie brilliant blue (R-250) (Sigma-Aldrich, San Luis, Misuri, USA). The protein concentration was quantified by the colorimetric method in accordance with Lowry et al. [59] employing bovine serum albumin as the standard. For the structural and functional trials performed in this work, the TvG6PD::6PGL protein was digested with TEVP protease (previously purified) to remove the additional amino acid residues corresponding to the (His)6-tag sequence located in the N-terminal region of the G6PD protein. The digestion was performed as previously reported by Ramírez-Nava et al. [44]. The purified recombinant protein was preserved in 50% glycerol (*v*/*v*) and stored at –70 °C.

### 3.6. Functional Analysis of TvG6PD::6PGL Protein

#### 3.6.1. Native Status of the Fused TvG6PD::6PGL Protein

The oligomeric state of the TvG6PD::6PGL protein in solution was determined by size exclusion chromatography. For this, the TvG6PD::6PGL protein and the gel filtration standards (Bio-Rad, Hercules, CA, USA) were loaded to a Sephacryl 400 (16/60) gel filtration column (GE Healthcare, Chicago, Illinois, USA) previously equilibrated with phosphate buffer (50 mM pH 7.35) and coupled to an AKTA pure FPLC system (GE Healthcare, Chicago, Illinois, USA). The protein and the gel filtration standards were eluted from the column using the same phosphate buffer with a flow rate of 0.3 mL/min, monitoring the absorbance at 280 nm (mUA). Both assays were performed in triplicate.

#### 3.6.2. Effect of Temperature and pH on TvG6PD::6PGL Activity

The effect of temperature on the activity of theTvG6PD::6PGL protein was determined by a thermal inactivation assay. The protein concentration was adjusted to 0.2 mg/mL in buffer T (100 mM Tris-HCl buffer pH 8.0, 0.01 M MgCl_2_). Next, the protein was incubated ( 20 min) at temperatures ranging from 35 °C to 60 °C. After incubation, the residual activity of the TvG6PD::6PGL enzyme was determined spectrophotometrically at 340 nm [23,44,47,60,61], and was expressed as a percentage of the residual activity. The enzyme activity incubated at 35 °C was set to 100%.

While, the effect of pH on the activity of the TvG6PD::6PGL protein was assessed by measuring the activity of G6PD at pH values ranging from 4.0 to 10.0 using different buffer systems (Mcllvaine buffer (pH 4.0–6.0), 50 mM MES buffer (pH 6.0–6.75), 50 mM HEPES buffer (pH 6.75–8.0), 50 mM Tris buffer (pH 8.0–9.0), and glycine (pH 9.0–10), as previously reported [23,43]. The non-enzymatic reduction of NADP^+^ in the assay was measured at each pH value and subtracted from the experimental points. Thermal inactivation and pH assays were performed in triplicate.

#### 3.6.3. Kinetic Characterization of the TvG6PD::6PGL Enzyme

The kinetic parameters of the G6PD region of the recombinant TvG6PD::6PGL protein were determined spectrophotometrically as previously reported by Gómez-Manzo et al. [62]. The activity assay was performed with the addition of 1 µg total pure TvG6PD::6PGL protein in a cuvette with a final volume of 1 mL. The steady-state kinetic for G6P substrate were obtained from the initial velocity data by varying the concentration from 0 to 800 µM, while the NADP^+^ substrate (second substrate) was fixed at 150 µM (saturating concentration). The steady-state kinetic of the NADP^+^ substrate was obtained from the initial velocity data by varying the concentration from 0 to 300 µM, while the G6P substrate remained saturated (1 mM). The initial velocities obtained for each concentration were fitting the data to the Michaelis–Menten equation via non-linear regression calculations [34], and the steady-state kinetic parameters, K_m_, *k_cat_*, and *V_max_*_,_ were obtained. All the assays were performed in triplicate.

### 3.7. Evaluation of the Stability of the TvG6PD::6PGL Protein by the NADP^+^ Molecule

#### 3.7.1. Thermal Inactivation Analysis

To evaluate the effect of the NADP^+^ molecule on the stability of the TvG6PD::6PGL protein, we performed a thermal inactivation assay in the absence and presence of the NADP^+^ molecule as previously reported by Morales-Luna et al. [45]. The protein was adjusted to 0.2 mg/mL in buffer T and incubated with three different concentrations of the NADP^+^ molecule (10, 100, and 500 μM) for 20 min in a T100 thermal cycler (Bio-Rad, Hercules, CA, USA) using a temperature gradient in a range from 37 to 60 °C. Next, the residual activity was measured spectrophotometrically at 340 nm, and the *T*_50_ value was determined. The residual activity at the three different concentrations of the NADP^+^ molecule measured at 37 °C was fixed as 100%. All thermal inactivation tests were performed in triplicate.

#### 3.7.2. Circular dichroism (CD) of TvG6PD::6PGL protein

To determine if the interaction of the NADP^+^ molecule caused a structural change in the integrity of the secondary structure of the TvG6PD::6PGL protein, the protein was analyzed by circular dichroism (CD) in a spectropolarimeter (Jasco J-810^®^, Inc., Easton, MD, USA) [61]. The TvG6PD::6PGL protein at 0.2 mg/ mL was incubated with the same NADP^+^ concentrations used for the structural stability test (10, 100, and 500 μM) for 2 h at 37 °C in a phosphate buffer. After incubation time, the spectrum ellipticity of the protein in the far UV region (200–260 nm) was recorded following changes in the α–helices and β–sheets at wavelengths of 222 and 208 nm, respectively. The blank emission spectrum (phosphate buffer) was subtracted from all spectroscopic readings. The curves were plotted using the Origin program. The experiment was performed in duplicate at 25 °C.

#### 3.7.3. Analysis of the Thermal Stability of the TvG6PD::6PGL Protein

To confirm that the interaction between the NADP^+^ molecule and TvG6PD::6PGL produced a protective effect against denaturalization by temperature, we evaluated the thermal stability of the recombinant TvG6PD::6PGL protein by CD monitoring the changes of the α–helices at 222 nm in their native conformation. For this purpose, the protein was adjusted to 0.2 mg/mL in 50 mM phosphate buffer with three different concentrations of NADP^+^ (10, 100, and 500 µM) over 2 h at 37 °C. The molar ellipticity was subsequently measured in a spectropolarimeter (Jasco J-810^®^, Inc., Easton, MD, USA) using temperature scans ranging from 30 to 90 °C. The data were processed in the Origin Lab program and fit to the Boltzmann sigmoidal equation to calculate the *T_m_* values [46].

#### 3.7.4. Susceptibility of the TvG6PD::6PGL Protein to Trypsin Digestion

The effect of the NADP^+^ molecule on the stability of the TvG6PD::6PGL protein was determined by the susceptibility to trypsin digestion without and with 1 K_m_ (27 µM) of NADP^+^ molecule. The TvG6PD::6PGL protein was adjusted to a final protein concentration of 0.2 mg/mL with buffer T and incubated with different trypsin concentrations in a range of 0 to 0.06 mg/mL at 37 °C for 2 h. Later, the residual activity was measured as previously reported [44,45,47] and expressed as a percentage of the activity of the enzyme without trypsin. Furthermore, we evaluated the residual activity during time-course inactivation (from 0 to 90 min) after incubating the TvG6PD::6PGL protein with a fixed concentration of trypsin (IC_50_ value = 0.01 mg/mL). The proteolytic digestion reaction was arrested by the addition of PMSF 5 mM, and the residual activity was measured each 2 min and expressed as a percentage of the activity of the same enzyme without trypsin.

#### 3.7.5. Susceptibility of the TvG6PD::6PGL Protein to Guanidine Hydrochloride (Gdn-HCl)

The effect of the NADP^+^ molecule on the recombinant TvG6PD::6PGL protein was analyzed in the presence of Gdn-HCl to assess if this molecule provides stability in the enzyme and thus confers higher resistance to denaturation. The TvG6PD::6PGL protein was adjusted to 0.2 mg/mL in buffer T and incubated with different concentrations of Gdn-HCl (0–1 M) and distinct NADP^+^ concentrations (10, 100, and 500 µM) over 2 h at 37 °C as previously noted [43,45,47]. Then, the residual activity was measured by spectrophotometrically monitoring the production of NADPH at 340 nm. The residual activity without Gdn-HCl was fixed as 100%.

### 3.8. Structural and Spectroscopic Characterization

#### Determination of the Ligand Dissociation Constant (K_d_) of Structural NADP^+^

To determine the ligand dissociation constant (*K_d_*) of structural NADP^+^, first the protein was dialysate and adjusted to 0.1 mg/mL and titrated with different NADP^+^ concentrations (from 0 to 1000 μM) and the intrinsic fluorescence was recorded from 310–500 nm in a Perkin–Elmer LS-55 fluorescence spectrometer (Perkin Elmer, Wellesley, MA, USA) using an excitation wavelength at 295 nm, with 5 and 15 nm slits for excitation and emission, respectively [45,49]. The final spectrum for each concentration of NADP^+^ was the average of five scans, and each spectrum was subtracted from the spectra of the blank (without protein plus NADP^+^). The *K_d_* was determined by plotting the maximum fluorescence intensity versus NADP^+^ concentration and adjusted to non-linear regression calculations with the equation for a single binding constant using the Origin program.

## 4. Conclusions

In this work, we reported the cloning, purification, and biochemical characterization of the fused G6PD::6PGL protein from the parasite *Trichomonas vaginalis*. The purified protein showed a molecular mass of 81.6 KDa per monomer, but we found this protein in the form of a tetramer; this differs from the other reported fused proteins, such as the G6PD::6PGL of *Giardia lamblia*, which are phylogenetically similar and feature observable dimeric and tetrameric forms. We also determined the kinetic parameters of the TvG6PD::6PGL protein for the G6P and NADP^+^ substrates and observed a higher catalytic constant value compared to the G6PDs from other parasites. In addition, the increase in resistance to temperature and thermostability in the presence of the NADP^+^ molecule suggests the existence of a structural NADP^+^ domain, which has been experimentally demonstrated in the same way as the domain present in the *G. lamblia* G6PD enzyme. Thus, this site likely began to appear in eukaryotic enzymes and has remained throughout their evolution, affording the enzyme greater stability and thus facilitating continuity of PPP. These results suggest new approaches for the study of the G6PD enzyme from *T. vaginalis* (and of other parasites that present fused proteins), which will facilitate the specific pharmacological objective to improve the efficiency of existing medications for the treatment of trichomoniasis.

## Figures and Tables

**Table 1:** Steady-state kinetic parameters of the fused TvG6PD::6PGL and the other G6PDs previously reported.

G6PD from Organism	K*_m_* G6P (mM)	K*_m_* NADP^+^ (mM)	*V*_max_ (µmol·min^−1^·mg ^-1^)	*k*_cat_ (s^−1^)	Reference
*T. vaginalis*	0.21	0.027	108.6	147	This study
*G. lamblia*	0.018	0.013	11.5	31.8	[23]
*P. falciparum*	0.019	0.006	5.2	8.6	[22]
*P. vivax*	0.080	0.014	5.6	6.7	[41]
*T. cruzi*	0.077	0.0016	NR	53.6	[40]
*T. brucei*	0.138	0.035	740.1	NR	[43]
*Homo sapiens*	0.038	0.007	160.1	230.3	[42]

NR = Data not reported.

## Supplementary Materials

Supplementary materials can be found at https://www.mdpi.com/1422-0067/21/14/4831/s1. Figure S1. Oxidative phase of pentose phosphate pathway (PPP) from *Trichomonas vaginalis*. Figure S2: Sequence obtained from the G6PD gene fused with *T*. *vaginalis* 6PGL. Figure S3. Structural superposition of the TvG6PD::6PGL model before and after energy minimization using the YASARA force field. Figure S4: Secondary structure. Supplementary Table S1: Summary of the purification of the recombinant TvG6PD::6PGL protein. TvG6PD::6PGL model obtained from Phyre2 (TvG6PD-6PGL_Phyre2.pdb). TvG6PD::6PGL model minimized (TvG6PD-6PGL_Minimized.pdb).
