# Supplementary material for: Characterizing the Fused TvG6PD::6PGL Protein from the Protozoan Trichomonas vaginalis, and Effects of the NADP+ Molecule on Enzyme Stability
Source: Int J Mol Sci. 2020 Jul 8;21(14):4831. doi: 10.3390/ijms21144831 (PMC7402283; doi:10.3390/ijms21144831)
Supplement: Supplementary file 1 [file ijms-21-04831-s001.zip › Supplementary Materials/Figure S1.pdf]

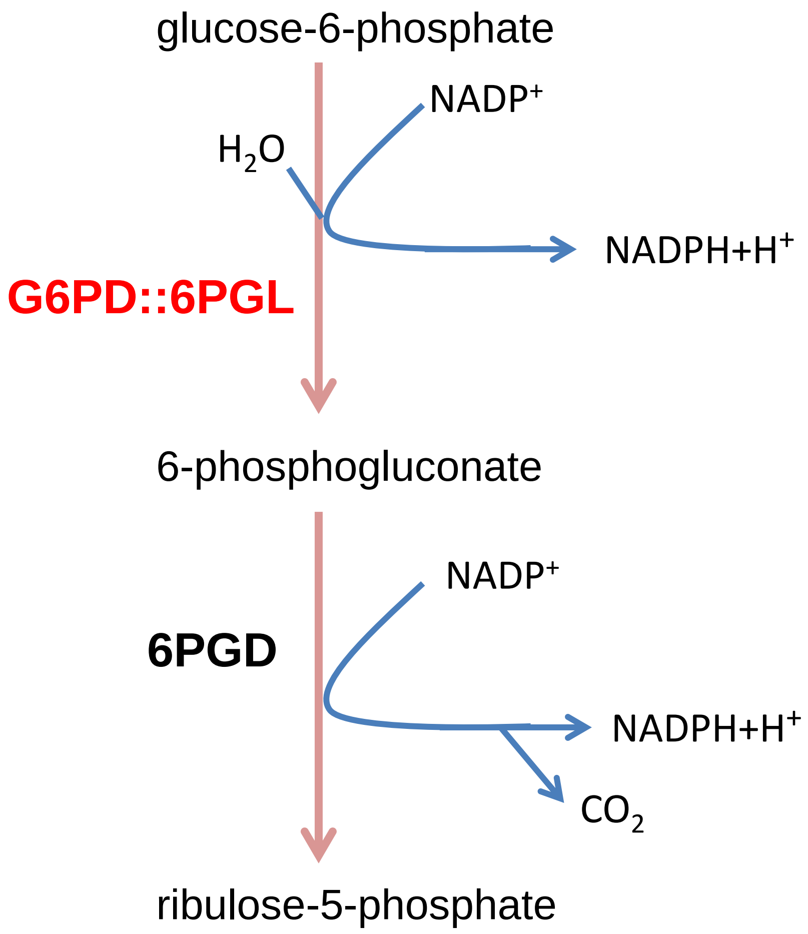

Figure S1. Oxidative phase of pentose phosphate pathway (PPP) from *Trichomonas vaginalis*. A schematic representation of oxidative phase of PPP in organisms with fused Glucose-6-phosphate dehydrogenase-6-Phosphogluconolactonase (G6PD::6PGL) enzyme.
