# Supplementary material for: Characterizing the Fused TvG6PD::6PGL Protein from the Protozoan Trichomonas vaginalis, and Effects of the NADP+ Molecule on Enzyme Stability
Source: Int J Mol Sci. 2020 Jul 8;21(14):4831. doi: 10.3390/ijms21144831 (PMC7402283; doi:10.3390/ijms21144831)
Supplement: Supplementary file 1 [file ijms-21-04831-s001.zip › Supplementary Materials/Figure S2.pdf]

atg act tct acc ttt tac gaa cct ttg aaa aat aag aag ata gca gca gtc atc ttt ggt
M T S T F Y E P L K N K K I A A V I F G
gcc tca ggt gat ctc tca atg aga atg ctt att cct tca ctt gaa tca atc tca ttg tac
A S G D L S M R M L I P S L E S I S L Y
gat cct ttc cac gaa gga aca atg atc atc ggc gtt gct agg tca aaa ttc act gat gaa
D P F H E G T M I I G V A R S K F T D E
gaa ctc cat gct aaa att aag aaa tct gtt att caa ttc tca cgt ctc cac cag gga tgt
E L H A K I K K S V I Q F S R L H Q G C
gat gat gaa aac agc gaa aca cca tgc aca gtc cca gat gag ttc tta aag aag atc aga
D D E N S E T P C T V P D E F L K K I R
tat atc tct ggt ggc tac gat gac ccc aat act tac caa gcc ctt aag aaa ttg atc gat
Y I S G G Y D D P N T Y Q A L K K L I D
gag aac caa ttc gaa ggc gtc atc gtt tat ttc gcc aca cct cct tct ctt ttc cat gtt
E N Q F E G V I V Y F A T P P S L F H V
atc tcc gat aac ctt aag agc aac ggc ctc aca tca aac gct cag cgc tgg att aga atc
I S D N L K S N G L T S N A Q R W I R I
att atc gaa aag cct ttc ggc aca tcc tac aag aca gct ctt gaa ctc aac gat tca ctt
I I E K P F G T S Y K T A L E L N D S L
cat agc aag ttc tcc gaa gaa gat ctt tat cga att gat cac tat ctc gct aaa gaa act
H S K F S E E D L Y R I D H Y L A K E T
gtt atg aac att ttc aca ttc cgt tgg gga aac acc atc tgg gaa cca ctt tgg aac aga
V M N I F T F R W G N T I W E P L W N R
aac tat atc tcc cat gtc gaa atc act gtc gct gaa gca gtt ggt gtc gag aat cgt atc
N Y I S H V E I T V A E A V G V E N R I
gga tat tac gaa ggc gtt tcc gtt atc aga gat atg atc cag aac cac ctt ctt cag atg
G Y Y E G V S V I R D M I Q N H L L Q M
ctt tcc att gtt gct atg gag cca cca tcc gaa atg aac gcc aag gcc atc aga gat gag
L S I V A M E P P S E M N A K A I R D E
aaa tta aag gtt ctc aga gcc atc aga gct gtt cag aag gat gac gtc gtc ctt ggt caa
K L K V L R A I R A V Q K D D V V L G Q
tat att ggc tac aga gag cac gaa ggt gtc cca gaa gat gct aca aca cca aca ttt gcc
Y I G Y R E H E G V P E D A T T P T F A
ttc atc aga ttc ttc att gac aac tgg aga tgg cag ggt gtc cca ttc tac atc tgc tca
F I R F F I D N W R W Q G V P F Y I C S
ggc aag gct ctc aag gag aag agg tct tcc atc aag tta gtc ttc aga gac att cct cat
G K A L K E K R S S I K L V F R D I P H
gcc tta ttc ggt gat tcg aca atc aac aaa cct aac tac ttg gag atc aag gtc cag cca
A L F G D S T I N K P N Y L E I K V Q P
aag gaa ggt atc atc ctc aag cag cac gtc aag gtc cca ggt atc ggc ttg aga aca gat
K E G I I L K Q H V K V P G I G L R T D
gtc atc cca tta tcc ttc tat tac aag gac aag ttc gga gac aat gct ttg gca ggt gcc
V I P L S F Y Y K D K F G D N A L A G A
tac gaa cgt gtt atc ctc gat gcc atc cac gga gat caa tcc ttc ttc cca cgt tcc gat
Y E R V I L D A I H G D Q S F F P R S D
gaa atc gaa gaa tgc tgg aag att gtc gat cca ctt ctc aaa gac aag tac cca gag atc
E I E E C W K I V D P L L K D K Y P E I
att cct tac gcc aag aaa ctc gat att ggt gtc ggc atg atc aag aga aac aag aag aac
I P Y A K K L D I G V G M I K R N K K N
ctc gca cag aga gtt gtt ttc aga aca cca tca gaa tta att cat tca gtc aca gaa caa
L A Q R V V F R T P S E L I H S V T E Q
atc act cgt ctt att aca gag gct att tct gag aga gga atc tgc aac atc gct ctt tca
I T R L I T E A I S E R G I C N I A L S
gga ggt caa aca cca aag cca att tac tca tta ctc tct aca aca cca tac gct gcc aga
G G Q T P K P I Y S L L S T T P Y A A R
att gat ttc agc aaa gtc cac atc tgg ttt gtt gat gag cgc tgt gtt cca cca gag cat
I D F S K V H I W F V D E R C V P P E H
aac ctc tcg aac tac cac atg atc aac gaa tct ctt ctc aga ttc atc aag att cca gaa
N L S N Y H M I N E S L L R F I K I P E
gag aac atc cac aga atc aga ggc gaa atc aac gct gaa gat gca gct aag gaa tac tcc

| | | | | | | | | | | | | | | | | | | | |
|---|---|---|---|---|---|---|---|---|---|---|---|---|---|---|---|---|---|---|---|
| E | N | I | H | R | I | R | G | E | I | N | A | E | D | A | A | K | E | Y | S |
| gac | gag | atc | atc | aaa | cac | ttt | gga | aca | gag | ata | cca | tca | ttc | gat | atc | tgc | ttg | ctt | gga |
| D | E | I | I | K | H | F | G | T | E | I | P | S | F | D | I | C | L | L | G |
| atg | ggt | aag | gaa | gga | cac | aca | gct | tcc | ctc | ttc | cca | ggt | tca | cca | gca | atc | cag | gat | aag |
| M | G | K | E | G | H | T | A | S | L | F | P | G | S | P | A | I | Q | D | K |
| gaa | agc | ctc | gtc | atc | ggt | gtc | ttc | gtt | cct | cac | gtt | aag | atg | ttc | aga | gtc | aca | ttc | gga |
| E | S | L | V | I | G | V | F | V | P | H | V | K | M | F | R | V | T | F | G |
| agg | aag | atc | atc | aac | aac | tct | aga | aac | atc | atg | ttc | atc | atc | act | gac | acg | gac | aag | aat |
| R | K | I | I | N | N | S | R | N | I | M | F | I | I | T | D | T | D | K | N |
| gag | atc | att | gac | ttg | gtc | ttc | aat | gct | cct | tac | gtc | cca | gcg | atg | atc | cct | gcc | cag | gtt |
| E | I | I | D | L | V | F | N | A | P | Y | V | P | A | M | I | P | A | Q | V |
| gtt | aca | gct | aag | gct | aaa | gat | gga | aag | ctt | tat | tgg | ttc | cat | gtc | gta | taa | | | |
| V | T | A | K | A | K | D | G | K | L | Y | W | F | H | V | V | * | | | |

Figure S2. Sequence obtained from the G6PD gene fused with *T. vaginalis* 6PGL. Nucleotide sequence and amino acid deduction of the fused protein. Amino acids corresponding to the G6PD (red) and 6PGL (blue) protein as reported in the conserved domine database (CDD) with reference number 235579 and 238694, respectively.
