# Supplementary material for: Characterizing the Fused TvG6PD::6PGL Protein from the Protozoan Trichomonas vaginalis, and Effects of the NADP+ Molecule on Enzyme Stability
Source: Int J Mol Sci. 2020 Jul 8;21(14):4831. doi: 10.3390/ijms21144831 (PMC7402283; doi:10.3390/ijms21144831)
Supplement: Supplementary file 1 [file ijms-21-04831-s001.zip › Supplementary Materials/Figure S3.pdf]

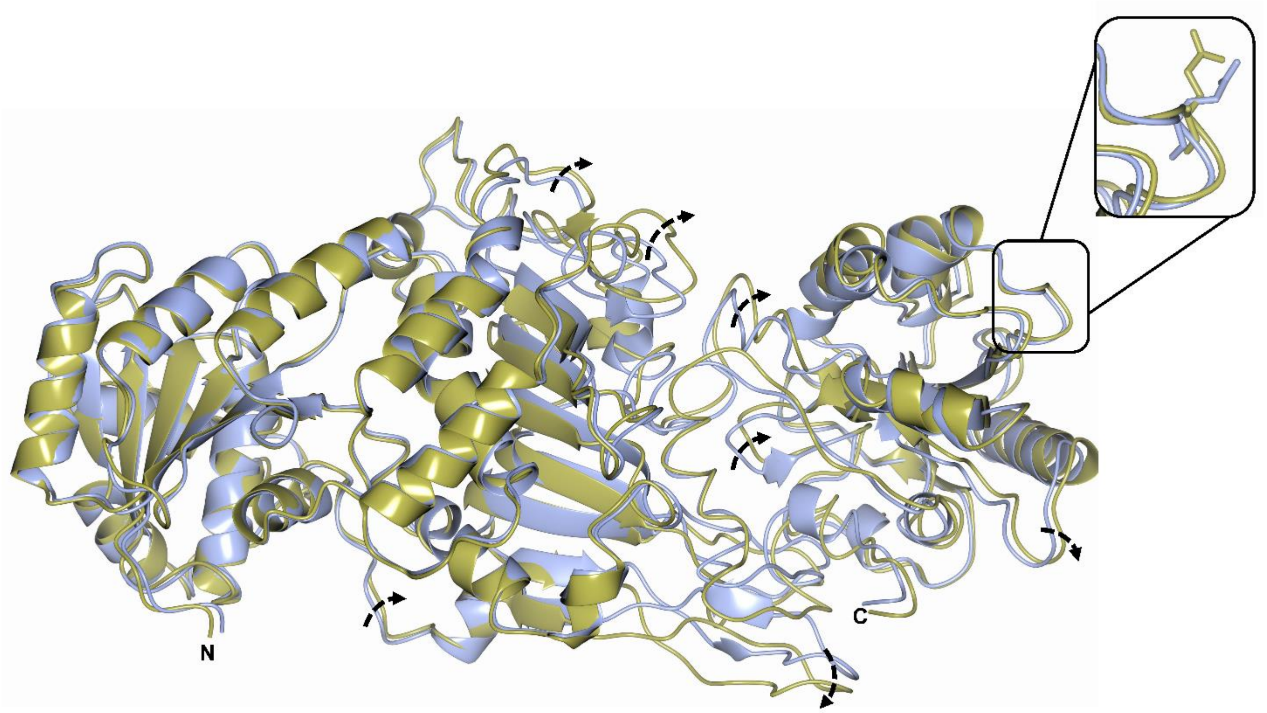

Figure S3. Structural superposition of the TvG6PD::6PGL model before (ice blue) and after (gold) energy minimization using the YASARA force field. Note the structural changes in some unstructured loops. Right inset: Close-up view showing a side-chain conformational change after energy minimization.
