# Supplementary material for: Characterizing the Fused TvG6PD::6PGL Protein from the Protozoan Trichomonas vaginalis, and Effects of the NADP+ Molecule on Enzyme Stability
Source: Int J Mol Sci. 2020 Jul 8;21(14):4831. doi: 10.3390/ijms21144831 (PMC7402283; doi:10.3390/ijms21144831)
Supplement: Supplementary file 1 [file ijms-21-04831-s001.zip › Supplementary Materials/Figure S4.pdf]

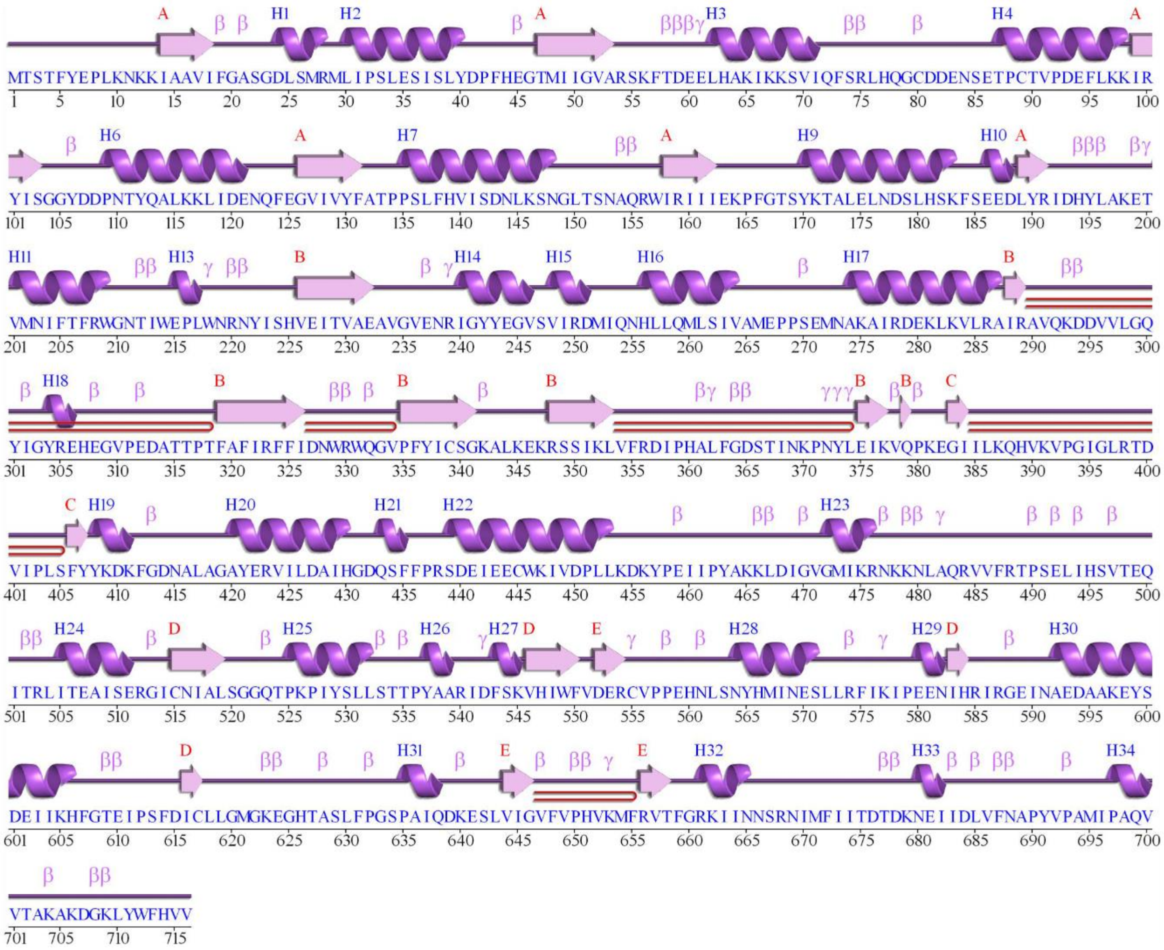

Figure S4. Secondary structure motif of Tv G6PD::6PGL protein from *T. vaginalis*. Secondary structure elements are shown as α–helices and β–sheets. The numbers indicate the corresponding amino acid residues.
