# Supplementary material for: Characterizing the Fused TvG6PD::6PGL Protein from the Protozoan Trichomonas vaginalis, and Effects of the NADP+ Molecule on Enzyme Stability
Source: Int J Mol Sci. 2020 Jul 8;21(14):4831. doi: 10.3390/ijms21144831 (PMC7402283; doi:10.3390/ijms21144831)
Supplement: Supplementary file 1 [file ijms-21-04831-s001.zip › Supplementary Materials/Table supplementary 1.pdf]

Supplementary file

Table supplementary 1. Summary of the purification of the recombinant TvG6PD::6PGL protein.

**Step**	**Total protein (mg)**	**Specific activity ($IU \cdot mg^{-1}$)**	**Total activity ($\mu mol \cdot min^{-1} \cdot mg^{-1}$)**	**Yield (%)**
Crude extract	106	1.8	190.8	100
Ni Sepharose	12.4	108.6	1342	47.5
